# Tumor Progression, Parallel Mechanisms and Therapeutic Targets

**DOI:** 10.3390/cancers18152518

**Published:** 2026-08-06

**Authors:** Leif Håkansson, Pontus Dunér, Annika Håkansson

**Affiliations:** 1 Division of Clinical Tumorimmunology, Department of Oncology, University Hospital of Linkoping, 581 85 Linkoping, Sweden; 2 Therim Diagnostica AB, 236 37 Höllviken, Sweden; 3 Department of Clinical Sciences Malmö, Lund University, 205 02 Malmö, Sweden; pontus.duner@redoxis.com; 4 Department of Oncology, Uppsala University Hospital, 751 85 Uppsala, Sweden; annika.hakansson@akademiska.se

**Keywords:** IL-6-inducing factor (IL-6IF), interleukin-6, hypoxia, HIF-1α, STAT3, tumor microenvironment, proteolysis, immune dysregulation, metabolic reprogramming, cancer immunotherapy

## Abstract

Cancer progression is driven by early dysregulation of the immune system and tumor-intrinsic mechanisms. Hypoxia, lactate accumulation and IL-6 signaling induce highly overlapping tumor-promoting effects, including angiogenesis, epithelial–mesenchymal transition, metastasis, immune evasion and treatment resistance, suggesting that these pathways interact and amplify one another. This parallel activation complicates therapeutic targeting, as inhibition of one pathway may be compensated for by another. Increased proteolytic activity emerges early during tumor development and profoundly alters immune regulation. We recently identified a protease-generated albumin fragment, the IL-6-inducing factor (IL-6IF), which triggers pathological IL-6 production. IL-6 in turn enhances both HIF-1α expression and nuclear translocation, promotes glycolysis and lactate production, and forms positive feedback loops with STAT3 and multiple signaling pathways. Together, these mechanisms integrate into a self-sustaining IL-6/HIF-1α/STAT3 axis that drives tumor progression and suppresses anti-tumor immunity. The strong overlap among IL-6, its enhancing loops and hypoxia-driven mechanisms highlights IL-6 as a central regulator of metabolic and immunological reprogramming in cancer. However, a broad IL-6 blockade can impair physiological immune function. Selective inhibition of IL-6IF offers a novel strategy to prevent pathological IL-6 production while preserving physiological IL-6-dependent immune function required for effective tumor control. Reducing pathologically enhanced IL-6 synthesis by targeting IL-6IF, therefore, might represent a potential therapeutic approach to disrupt multiple tumor-promoting pathways simultaneously and may thereby improve responsiveness to cancer immunotherapy.

## Simple Summary

The immune system has the capacity to control cancer, but in the presence of malignant tumors and chronic inflammation, a cascade of dysregulated mechanisms takes over. Immune-mediated cancer control is gradually replaced by mechanisms that promote tumor development. Interactions between these mechanisms make it difficult to identify drugs that effectively block these pathways to restore immune-mediated cancer control. Potentially efficient cancer therapies may lose their efficacy because parallel tumor-promoting mechanisms can compensate when a single pathway is blocked. This paper describes the need to further explore and understand the interaction between these parallel mechanisms in order to find drugs or combinations of drugs that can re-establish cancer control. Early tumor growth leads to increased protein degradation and oxygen deprivation (hypoxia), a microenvironment that induces the production of a regulatory cytokine called interleukin-6, which strongly promotes cancer development. A novel monoclonal antibody candidate that selectively reduces pathological IL-6 production in cancer is also described.

## 1. Introduction

### 1.1. Cancer Progression

Cancer progression depends on deranged tumor cell characteristics and the failure of the immune system to eradicate malignant cells. The capacity of the immune system to control and even cure cancer has been demonstrated during recent decades by treatment with checkpoint inhibitors and CAR-T cells. However, still only a minority of cancer patients benefit from immunotherapy. Multiple immune mechanisms are dysregulated in cancer, creating a pro-tumorigenic milieu. Different types of immune cells acquire immunosuppressive anti-tumor re-activity, e.g., tumor-associated macrophages, myeloid-derived suppressor cells, regulatory T-cells and immature dendritic cells [1]. Immunosuppression is further regulated by the cytokine and chemokine network.

The mechanisms that initiate an effective anti-tumor immune response are well understood: cytotoxic T cells and natural killer cells recognize and lyse tumor cells [2,3]. By contrast, the early events that dysregulate the immune system, leading to immunosuppression and therapeutic resistance, remain poorly understood.

### 1.2. Primary and Second-Generation Immunoregulatory Mechanisms

In advanced tumors, an extensive, complex network of cytokines, growth factors and suppressive immune cells is involved in the dysregulation of the immune system to create a hostile, pro-tumorigenic milieu resulting in tumor progression and resistance to treatment. What are the primary mechanisms initiating this cascade of interactions? The majority of tumor-promoting mechanisms found in advanced tumors are a consequence of multiple interactions. These secondary mechanisms develop once the dysregulatory cascade is initiated. Therapeutic strategies targeting such mechanisms, therefore, require multi-targeted interventions to achieve efficient tumor eradication. This treatment approach also necessitates reliable predictive tests to guide therapeutic response.

A large number of cancer drugs, directed against multiple targets, have been developed. Unfortunately, most of these drugs have limited efficacy. In order to overcome these short-comings, combinations of drugs are used, but generally with far from optimal efficacy. The number of possible drug combinations is enormous. The number of available patients is insufficient to conduct clinical studies to systematically evaluate and identify the most effective combinations. In order to identify the best possible drug combinations, extensive in vitro studies have been performed [4,5]. However, despite these extensive efforts, translating in vitro findings into clinically effective combination therapies remains a major challenge, largely due to the complexity and dynamic nature of tumor-associated immune dysregulation.

### 1.3. Parallel Regulatory Mechanisms

Analyzing the regulatory network in cancer is further complicated, as there are parallel mechanisms generating very similar effects on deranged tumor cell biology and dysregulation of the immune system. Hypoxia and enhanced proteolytic activity, which result in IL-6 and HIF production, appear early in tumorigenesis and are both of paramount importance in most mechanisms promoting tumor progression (Table 1 and Table 2).

This highlights the need to focus on careful exploration of the interaction of these fundamental mechanisms in order to identify targets with a fundamental, decisive impact on multiple tumor-promoting activities with a specificity not resulting in serious adverse events.

## 2. Tumor-Associated Antigens—Immune Complexes—Immunosuppression

In the tumorigenic process, some tumor molecules are overexpressed and new molecules appear due to mutations. These tumor-associated antigens, TAA, initiate an immune response, including auto-antibodies, e.g., anti-p53, -c-MYC and -MUC-1 [41,42]. This means that TAA and auto-antibodies are present in cancer patients as free antigens or free antibodies and as immune complexes. The characteristics of such complexes depend on the ratio between these constituents and the valency of the antigens.

Immune complexes binding to Fc receptors on immune cells can be critical in both activation and downregulation of immune responses [43] by modulating the production of cytokines, such as interleukin-6 [44], and inhibiting activation of naïve T-cells [45]. The occurrence and immunosuppressive activity of immune complexes in cancer is well recognized [46,47]. In addition, anti-tumor antibodies binding to Fc-receptors on effector cells such as NK-cells, can elicit Antibody Dependent Cellular Cytotoxicity.

Serum factors blocking anti-cancer immune reactivity were described several decades ago [48,49]. The immunosuppressive activity of the blocking factors in sera from patients and animals with progressing tumors was neutralized by sera from animals with regressing tumors [50,51], indicating an interaction between immune complexes in antigen or antibody excess. Further characterization of serum blocking factors supports that these factors are made up of immune complexes [52].

A new type of immunoregulatory serum factor partly bound in immune complexes was recently described. Immunoregulatory albumin neo-structures were generated by the degradation of serum albumin by a pathologically enhanced proteolytic activity in cancer patients. One of these serum factors with the capacity to induce IL-6 synthesis was found to be significantly increased in advanced cancer and was correlated with poor cancer-specific survival [53].

Taken together, a convincing documentation showed the immunoregulatory importance of serum factors other than cytokines in cancer. These serum factors have the capacity to act synergistically with immune cells, inducing immunosuppressive activity. Based on these results, it can be concluded that ex vivo studies with immune cells from cancer patients should contain autologous serum (serum factors) in order to adequately mirror the in vivo situation.

## 3. Hypoxia

**Figure 1:**
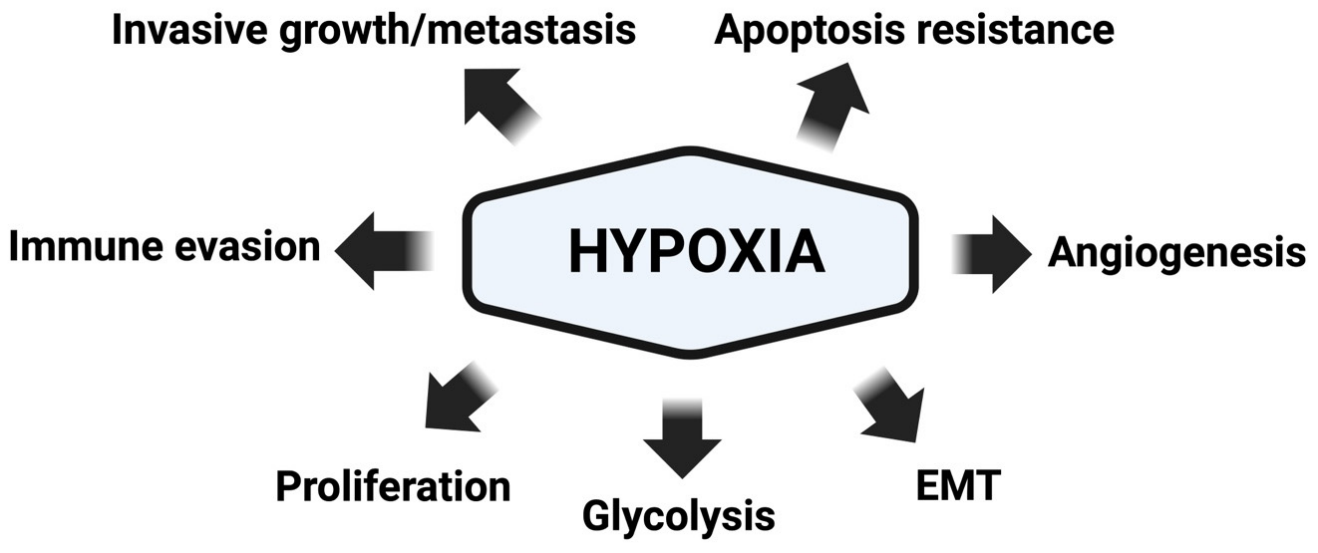
Hypoxia as a central driver of tumor progression. Hypoxia arises in solid tumors due to insufficient vascularization and dysfunctional tumor vasculature. Reduced oxygen availability activates hypoxia-inducible factors (HIFs), placing hypoxia at the center of a coordinated tumor-promoting response. Through HIF-dependent and HIF-independent mechanisms, hypoxia directly drives angiogenesis, proliferation, apoptosis resistance, and glycolysis. In parallel, hypoxia promotes epithelial–mesenchymal transition (EMT), leading to invasive growth/metastasis, and induces immune evasion by suppressing anti-tumor immune responses in the tumor microenvironment. Together, these hypoxia-driven processes cooperate to promote tumor progression and therapeutic resistance (Created in BioRender. Dunér P. (2026) https://BioRender.com/h42n006.).

Malignant tumors outgrow their oxygen supply at a size of just a few millimeters. Inadequate angiogenesis and the structurally abnormal vasculature of tumors further contribute to a hypoxic microenvironment. Hypoxia influences numerous aspects of tumor biology, including altered cellular metabolism, induction of angiogenesis, promotion of invasion and metastasis, and inhibition of apoptosis (Figure 1). In addition, hypoxia profoundly disrupts immune regulation, creating a TME that supports tumor progression [54]. Consequently, hypoxia is strongly associated with poor prognosis and resistance to non-surgical treatment modalities, such as radiotherapy, chemotherapy and immunotherapy.

The response to hypoxia is primarily mediated by hypoxia-inducible factor (HIF-1), a transcription factor composed of a labile HIF-1α subunit and a constitutive HIF-1β subunit [55]. HIF-1α binds to hypoxia-responsive elements in the promoter and enhancer regions of HIF-1 and activates transcription of numerous target genes (Table 1) of crucial importance for tumor progression, including angiogenesis, cancer stemness, cell motility, epithelial–mesenchymal transition, extracellular matrix remodeling, invasion, and metastasis, glucose and lipid metabolism and immune evasion [55,56].

**Figure 2:**
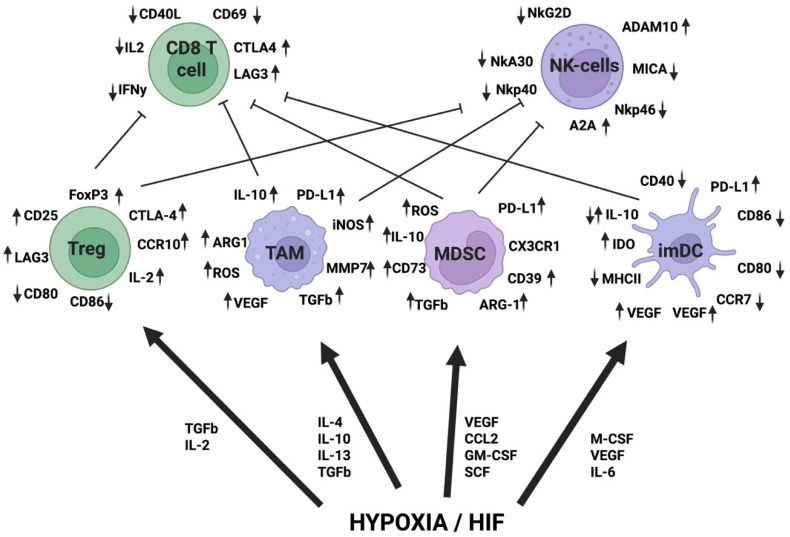
Hypoxia-induced regulation of immune cells in the tumor microenvironment. Molecules that are upregulated (↑) or downregulated (↓) under hypoxic conditions are indicated next to each cell type. Cytokines and soluble factors promoting the generation or activation of immunosuppressive cells are shown along the arrows (Created in BioRender. Dunér P. (2026) https://BioRender.com/h42n006.).

In hypoxia, immunosuppression is initiated and sustained by a network of soluble mediators and regulatory immune cells (Figure 2). As shown in Figure 2, HIF transcription factors regulate a large number of target genes expressed by different suppressor cells. The complexity of this dysregulation of the immune system is demonstrated by the expression of the same factors by different suppressor cells. Key anti-tumor effector cells, including T cells, NK cells, and dendritic cells, are inhibited, and immunosuppressive populations are actively recruited to the tumor microenvironment [55].

## 4. Lactate

As hypoxia and HIF play a major role in the induction of glycolysis, lactate and lactylation can be considered partly as mediators of dysregulation of the immune system in hypoxia. Tumor cells and immune cells adapt to a hypoxic microenvironment by shifting from oxidative phosphorylation to anaerobic and aerobic glycolysis [57]. The hypoxia-induced transcription factor HIF-1 enhances the expression of glycolytic enzymes, resulting in an increased production of lactate and acidification of the tumor microenvironment [58]. Lactate was, until recently, considered to be a waste product, but is now recognized as a signaling molecule that influences tumor cell biology directly, as well as indirectly through post-translational protein modifications such as lactylation. High levels of lactate in the tumor microenvironment are strongly associated with poor prognosis and resistance to therapy.

Lactate induces profound changes in tumor cell biology and modulates immune cell function by activating the GPR81 and MCT receptors. Tumor progression is promoted by stimulation of tumor cell growth, cell migration, angiogenesis, EMT, invasive growth and metastasis [59,60]. Because many of these effects overlap with those mediated by hypoxia and HIF-1 signaling, it remains challenging to distinguish between the direct consequences of HIF-1α–regulated gene expression and those driven by lactate through its receptors.

A high concentration of lactate, causing an acidic milieu, inhibits effector immune cells, including T cells and NK/NKT cells. The immunosuppressive milieu is further supported by inhibition of cytokine production, inhibition of T-cell proliferation [61], inhibition of antigen-presenting cells and upregulation of PD-L1. Simultaneously, the recruitment of suppressor cells, regulatory T cells (Tregs), myeloid-derived suppressor cells (MDSCs), and M2 macrophages is increased. Lactate promotes M2 polarization of macrophages and differentiation of MDSC [62]. Furthermore, lactate has been implicated in treatment resistance [63].

The complexity of the effects of lactate, with both pro- and anti-tumor activities, is shown as it can, in addition to the pro-tumor activities described above, increase the stemness of CD8+ T-cells and thereby enhance their anti-tumor activity [64,65].

### 4.1. Protein Lactylation

In addition to the direct immunoregulatory, as well as tumor-promoting effects of lactate, an enhanced production of this metabolite results in post-translational modification of proteins via lactylation. Histone lactylation increases tumor-infiltrating myeloid cells and polarizes tumor-associated macrophages (TAMs) toward the M2 phenotype expressing HIF-2α, ARG1, and VEGF [66]. However, histone lactylation can also mediate transcriptional repression of the macrophage inflammatory response [67]. Histone lactylation, H3K18la, was found to downregulate RARγ, which through upregulation of TRAF6, IL-6 and STAT3, creates a positive feedback loop for IL-6 production and tumor progression [68].

Histone lactylation has also been linked to treatment resistance, e.g., reduced efficacy of bevacizumab treatment of colorectal cancer [69], resistance to cisplatin in bladder cancer [70] and in oral squamous cell carcinoma [71]. In head and neck squamous cell carcinoma, the histone lactylation H3K9la correlates with the expression of IL-11, an IL-6 family member, and poor response to immunotherapy [72].

### 4.2. Proteases in Inflammation and Cancer

Tumorigenesis is closely linked to inflammation and upregulated proteolytic activity [73,74]. It is well established that a large number of proteases create a pathological proteolytic microenvironment in cancer. The proteolytic activity is upregulated at early stages of tissue damage, resulting in numerous protein fragments [75]. One of these fragments is an albumin neo-structure with the capacity to induce pathological IL-6 production, IL-6IF [53]. Protease-activated receptors (PARs) play a major role in cancer progression [76]. Extracellular matrix (ECM) degradation promotes tumor invasion and metastases. Caspases are essential in the cytolytic activity of immune cells [77]. Cathepsin D is of importance for the activation of LFA-1 and thereby the recruitment of effector T-cells. Proteolytic fragmentation of cytokines and chemokines modulates their activity [78,79]. Proteases activate TGFβ by cleaving the latent complex [80]. Shedding from the cell surface contributes to activation of TNF-α, and the shedding of the IL-6 receptor is a prerequisite for its binding to gp130 and the alternative trans-signaling mechanism of this cytokine. The protease thrombin was found to induce IL-6 production in epithelial cells and fibroblasts [81]. Serine proteases have the capacity to release IL-6 from monocytes [82].

### 4.3. Interleukin-6

Interleukin-6 (IL-6), a pleiotropic, pro- and anti-inflammatory cytokine, plays a central role in both physiological and pathological immunoregulation. An enhanced serum concentration of IL-6 is associated with poor prognosis in most types of cancer [83].

Under normal regulation of the immune system, synthesis of IL-6 is usually induced by damage-associated molecular patterns (DAMPs), pathogen-associated molecular patterns (PAMPs) or a cytokine signaling network. Several molecules belonging to these molecular patterns, DAMP or PAMP, induce IL-6 by interaction with the TLR and RAGE receptors [84,85,86], thus stimulating the inflammatory response.

The concept of the “Local Initiation Model” proposes that various types of tissue injury trigger proteolytic activity and inflammation [87,88,89]. The proteolytic activity is upregulated at early stages of tissue damage, resulting in numerous protein fragments [75]. As described here, an albumin fragment with the capacity to induce pathological IL-6 synthesis was discovered [53].

**Figure 3:**
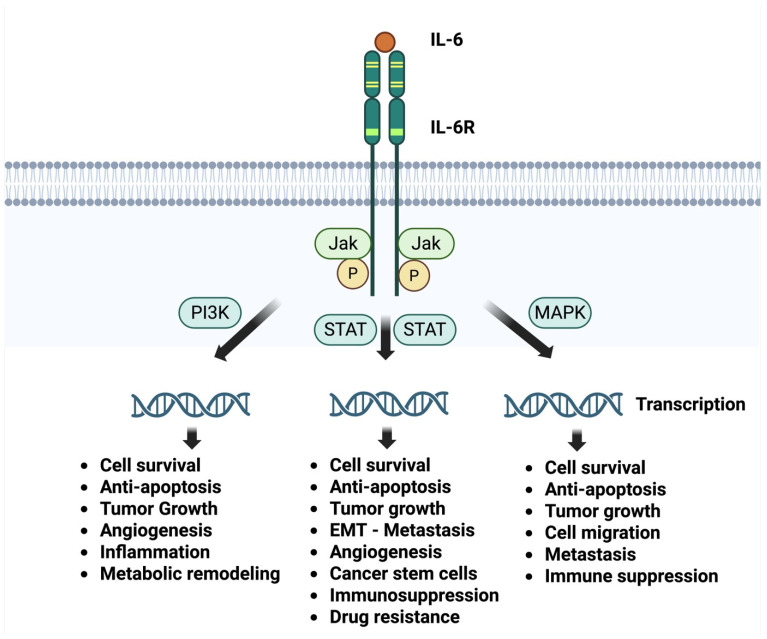
Different signal pathways for IL-6, STAT3, PI3K and MAPK, potentially activating different promotors of cancer progression (Created in BioRender. Dunér P. (2026) https://BioRender.com/h42n006.).

Interestingly, IL-6 acts as an enhancer of inflammation. Activation of several important signal transduction pathways (Figure 3) involved in tumor progression, such as PI3K/AKT/mTOR [90,91], Ras/MAPK [92,93], Wnt/β-catenin [94,95] and Notch [96,97], results in enhanced IL-6 production. Furthermore, the activity of several of these pathways, including the major signaling pathways of IL6, JAK/STAT3, PI3K/AKT and Ras/MAPK [98,99], can be activated by IL-6, which results in an IL-6-enhancing loop further promoting tumor progression.

The transcription factor NF-kappaB is a key regulator of inflammation and synthesis of IL-6 [100]. Similar to the situation with the signal transduction pathways described above, IL-6 also has the capacity to modulate the activity of NF-kappa B [101]. Synergistic interaction between STAT3 and NF-kappa B results in further amplification of the production of IL-6 and other cytokines, the IL-6 Amplifier mechanism described by Hirano [89].

**Figure 4:**
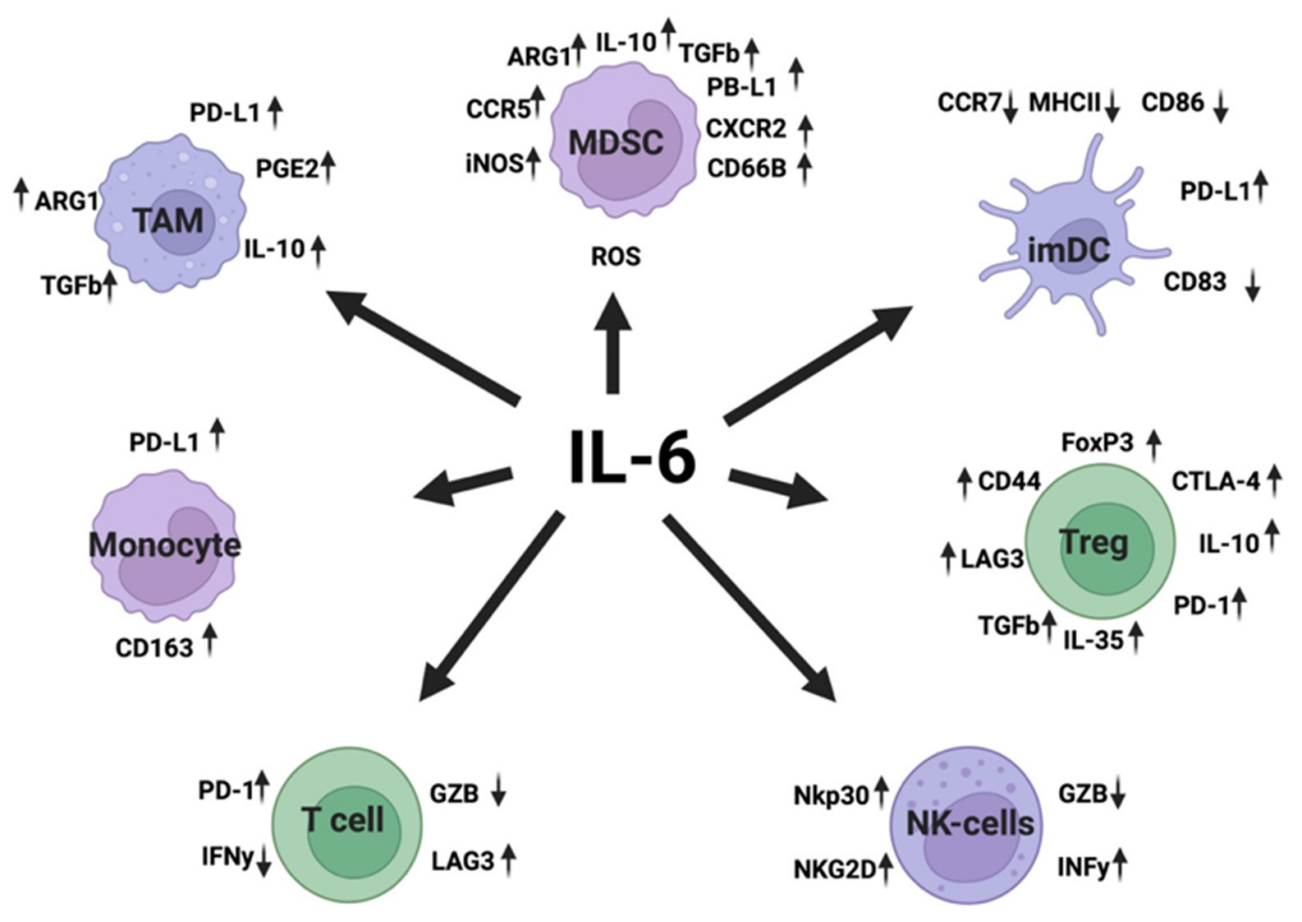
Modulation of immunosuppressive factors/mechanisms by IL-6 (Created in BioRender. Dunér P. (2026) https://BioRender.com/h42n006.).

IL-6 is of key importance in suppressing the anti-tumor reactivity of the immune system, e.g., supports T helper 2 cells (IL-4 production) and inhibits T helper 1 cells (IFNγ production), inhibits dendritic cell differentiation and function, decreases expression of MHC class II and CD86 by dendritic cells, thus attenuates T-cell stimulation, induces differentiation of M_2_ macrophages, induces MDSC differentiation and up-regulates PD-L1 expression (for details see Figure 4). IL-6 frequently acts in synergy with hypoxia (further discussed below).

The anti- and pro-inflammatory activity of IL-6 is mediated by classic or trans-signaling, respectively. In classic signaling, IL-6 is bound to its cellular receptor IL-6R, and then signal transduction occurs via binding to gp130. In trans-signaling, IL-6R is proteolytically released from receptor-positive cells, the soluble receptor binds IL-6, and this complex binds to gp130. IL-6R is expressed on a very limited number of cells (lymphoid and myeloid cells and hepatocytes), whereas gp130 is widely expressed on cells. IL-6 can thereby, via trans-signaling, influence a large number of different types of cells. Different biological effects due to signaling pathways have been further analyzed [102]. IL-6 plays a major role in the regulation of energy/glucose metabolism, classic signaling supports oxidative phosphorylation and trans-signaling shifts energy metabolism to anaerobic glycolysis [15].

IL-6 is critically involved in immunosuppression in cancer and therapeutic resistance in cancer. The need to suppress pathologically elevated IL-6 levels is widely recognized in the medical and scientific community to improve therapeutic control of diseases characterized by IL-6 overproduction. However, IL-6 inhibition has to be selective, leaving the normal activity of IL-6 and the normal function of the immune system intact, which is a prerequisite for ultimate cancer control.

The effect of serum factors on the pathological production of IL-6 was investigated in PBMC cultures containing autologous serum. These studies showed that factors present in cancer patient sera induced the synthesis of high levels of IL-6, even in patients with a serum concentration of IL-6 within the normal range. Pathological IL-6 production was also detected in early-stage patients with localized colorectal cancer or in patients with no clinically detectable disease, for example, those with radically resected stage III melanoma. Further analysis of these serum factors showed that proteolytic degradation of normal serum albumin results in immunoregulatory albumin neo-structures, one of which has the capacity to induce IL-6, the IL-6 inducing factor, IL-6IF. IL-6IF was found to be significantly elevated in advanced stages of cancer and was significantly correlated with reduced cancer-specific survival [53]. Regulation of IL-6IF is complicated by the presence of regulatory autoantibodies directed against this factor. Thus, IL-6IF and antibodies are bound in immune complexes, the composition of which depends on the relative abundance of IL-6IF and antibodies and the valency of albumin antigens. This mechanism offers a unique possibility to produce recombinant monoclonal antibodies for the selective removal of pathologically enhanced IL-6 production, leaving normal IL-6 production and the normal function of the immune system intact.

## 5. Stat3 Cancer

IL-6 can activate several signaling pathways, in particular, the JAK/STAT3, but also the PI3K/AKT and Ras MAPK pathways. The transcription factor STAT3 activates numerous genes that control key aspects of cell biology, promoting tumorigenesis and tumor progression. Constitutive activation of STAT3 is observed in more than 70 percent of human cancers and is related to poor clinical prognosis. Genes induced by activated STAT3 regulate tumor proliferation, stemness, survival, angiogenesis and immune evasion [103]. In addition to supporting tumor-promoting cellular characteristics, it plays a critical role in the development of immunosuppression and resistance to non-surgical treatment of cancer. STAT3 is up-regulated and activated by IL-6 and hypoxia. STAT3 activation is a prerequisite for the activity of the HIF-1α [104,105].

## 6. Synergy Between Hypoxia, IL-6 and Stat3 in Tumor Progression

As discussed above, hypoxia profoundly affects multiple aspects of tumor cell biology and contributes to immune dysregulation in cancer. Many of these mechanisms are also regulated by IL-6, suggesting an interaction between hypoxia, mediated by HIF-1, and IL-6 in the promotion of tumor progression (Table 1). This cross-talk adds additional regulatory components to tumor promotion and progression.

Enhanced protease activity appears early in inflammation and cancer, and as described above, proteolytic degradation of serum albumin results in an IL-6-inducing factor [53]. IL-6 was found to upregulate HIF-1α expression in both murine and human cell lines, and to promote tumor-associated immunosuppression. Treatment with an anti-IL-6 receptor antibody (a rodent analog to tocilizumab) suppressed tumor progression by down-regulating HIF-1α expression [106]. These findings highlight a reciprocal relationship between IL-6 and HIF-1α, reinforcing the hypoxia–IL-6–STAT3 axis as a central pathway driving tumor progression and immune evasion. The synergistic interaction between IL-6 and HIF-1αwas further demonstrated in ovarian cancer cell lines. IL-6 enhanced the expression, transcriptional activity and nuclear translocation of HIF-1α through STAT3 signaling. Furthermore, the IL-6/STAT3/HIF-1α feedback loop was also shown to contribute to the development of resistance to cisplatin in ovarian cancer cell lines [107]. Activation of STAT3 is a prerequisite for the activity of HIF-1α. In addition, the transcription of HIF-1a requires nuclear translocation, which is promoted by L-6 [107].

Conversely, HIF-1α was shown to stimulate the production of IL-6 as inhibition of HIF-1α reduced IL-6 and TNFα levels in a model with diabetic retinopathy [108]. These findings indicate a possible feedback loop that amplifies the pathological IL-6 production under hypoxic conditions.

Tumorigenesis is supported by the interaction between IL-6 and hypoxia mediated by HIF-1α. IL-6 promotes epithelial–mesenchymal transition (EMT), thereby facilitating invasive growth and metastasis in synergy with HIF-1α [109]. Micro-RNAs (miRNAs) are important regulators in cancer progression [110]. In ovarian cancer, IL-6 regulates tumor progression related to EMT, invasive growth and metastasis by down-regulating Let-7c or miR-200c via HIF-1α through the STAT3/HIF-1α signaling pathway [111].

In addition to these synergistic effects of hypoxia/HIF-1α and IL-6, numerous molecular mechanisms are described to be dysregulated by both pathways. However, these effects are usually not investigated in parallel within the same experiment (Table 2).

**Table 2:** Immunoregulatory molecules modulated by hypoxia, HIF-1α and IL-6.

Immunoregulatory Molecules	Hypoxia References	IL-6 References
ARG1	[112]	[113]
ROS	[114,115]	[116]
PD-L1	[117,118]	[119]
TGFβ	[120,121]	[122,123]
IL-10	[124]	[125,126]
VEGF	[127,128]	[129,130]
Adenosine	[131,132]	[133,134]
CD47	[135,136]	[137,138]
CD73	[139,140]	[141,142]
HLA-DR	[143]	[144]
CD80	[143]	[145]
CD86	[143,146]	[147]
EGFR	[148,149]	[150,151]

These comparisons indicate that inhibition of IL-6 may enhance anti-tumor immunity under hypoxic conditions.

In addition to illustrating the complexity of early regulation of cancer development, these examples on synergism between IL-6 and hypoxia/HIF-1α also highlight the possibility that additional interactions of this kind remain to be discovered. Such findings warrant comprehensive investigations that analyze multiple mechanisms in parallel.

## 7. IL-6 Regulation of Glycolytic Enzymes

**Figure 5:**
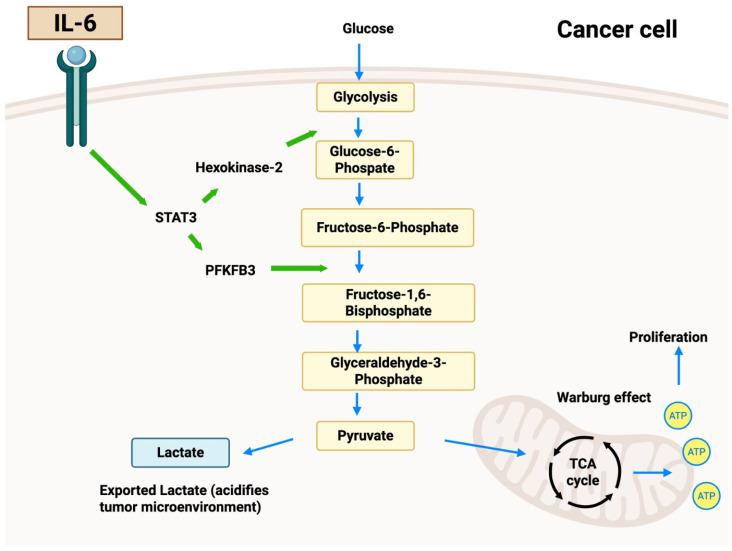
IL-6 promotes glycolytic reprogramming in cancer cells through STAT3-mediated regulation of HK2 and PFKFB3. Interleukin-6 activates the STAT3 signaling pathway, which upregulates the glycolytic enzymes HK2 and PFKFB3, enhancing glucose flux through glycolysis. This leads to increased production of pyruvate and lactate, supporting the metabolic needs of rapidly proliferating cancer cells. A fraction of pyruvate enters the TCA cycle in the mitochondria to generate ATP, while most is converted to lactate, contributing to the Warburg effect characteristic of cancer metabolism (Created in BioRender. Dunér P. (2026) https://BioRender.com/h42n006.).

Lactate plays a major role in promoting tumor progression; consequently, regulation of lactate production is crucial for controlling cancer growth. IL-6, through activation of STAT3, increases the expression of hexokinase 2 (HK2) and 6-phosphofructo-2-kinasefructose-2,6-bisphosphatase-3 (PFKFB3), two key enzymes that regulate glycolysis and consequently lactate production (Figure 5) [152].

PFKFB3 plays an important role in CRC tumorigenesis and resistance to immunotherapy [153]. The impact of PFKFB3 on colorectal cancer (CRC) was also demonstrated by its overexpression correlating with lymph node metastases, tumor emboli and TNM stage. SiRNA-mediated knockdown of PFKFB3 abolished the effect of IL-6 on CRC cells. Consistently, treatment with an anti-IL-6 receptor antibody efficiently down-regulated PFKFB3 expression [154].

Treatment of Raw264.7 cells with IL-6 increased both mRNA and protein levels of key glycolytic enzymes via AKT signaling, thereby promoting resistance to radiotherapy. Inhibition of AKT phosphorylation significantly reduced IL-6-induced HK2 expression and radio resistance [37].

In ovarian cancer, treatment with the antioxidant Resveratrol inhibited IL-6-promoted cancer progression by inhibiting glycolysis-mediated autophagy [155]. An IL-6 inhibitor, Bazedoxifene, not only suppresses IL-6-dependent cell survival, cell proliferation, and STAT3 activation but also decreases cellular glycolysis [156].

## 8. Discussion

The similarity in effects induced by hypoxia, lactate and IL-6 indicates that these mechanisms might interact, creating parallel and overlapping tumor-promoting effects. Identifying the most decisive upstream triggers is therefore critical for therapeutic targeting.

Hypoxia appears early in very small tumors, creating a milieu favoring metastasis by initiating mechanisms supporting epithelial–mesenchymal transition (EMT) and invasive growth. However, metastases usually do not develop at these early stages. Possibly, the combination of mechanisms necessary for metastasis has not evolved yet, or the immune system still has the capacity to eradicate metastatic cells, the elimination stage of the immunoediting process (immunosurveillance) [157].

Nevertheless, tumor aggressiveness generally increases with tumor size. Hypoxia and HIF activity increase with tumor stage and are closely associated with metastasis [158]. Similarly, proteolytic activity increases [159], thereby facilitating invasive growth and modulation of immune regulatory mechanisms. Degradation of albumin is well documented in cancer and is considered to be of major importance in order to supply tumor cells with energy and material for cell multiplication. Macropinocytosis, a fundamental mechanism in most cancers, results in the uptake and transportation of large amounts of albumin to lysosomes, where it is degraded [160]. Proteolytic degradation of serum albumin generates an IL-6-inducing factor (IL-6IF), linking proteolysis to pathological IL-6 production.

The serum concentration of IL-6IF correlates with tumor stage and poor survival [53]. IL-6IF has so far been demonstrated in breast cancer, colon cancer and lymphoma and autoantibodies against this factor have also been demonstrated in HNSCC. Taken together, this indicates that IL-6IF occurs in a large number of different types of cancer. This is in good agreement with increased serum concentration of IL-6 in advanced cancer and poor prognosis [83].

The central role of IL-6IF in pathological il-6 production in vivo is demonstrated by a significant inverse correlation between the serum concentration of autoantibodies directed against IL-6IF and the occurrence of IL-6 in colon cancer patients. Similarly, IL-6 production induced by serum factors can be completely inhibited by rabbit antibodies specifically directed against IL-6IF [53].

Our findings support a model in which hypoxia, lactate production and enhanced proteolytic activity generating IL-6IF act as parallel and interconnected initiators of immune dysregulation. IL-6IF induces IL-6 synthesis, which then reinforces HIF-1α and STAT3 signaling, forming a self-sustaining loop that promotes metabolic adaptation, tumor progression, and immune suppression. Similarly, IL-6 is a key regulator of glycolytic enzymes generating lactate.

While the immunoregulatory and tumor-promoting effects of IL-6, hypoxia, and lactate are well documented, the identification of IL-6IF as a proteolysis-derived inducer of IL-6 provides a novel mechanistic link among inflammation, proteolytic remodeling, and metabolic reprogramming. This places IL-6IF as a potential central regulator that connects tumor metabolism and immune escape.

Interconnected regulatory pathways create robust but less change-sensitive immune networks, as alterations in one pathway may be compensated for by another. This complicates therapeutic initiatives, since intended pharmacological modulation of one pathway may be counteracted by compensatory changes in others. Therapeutic efficacy, therefore, requires a profound understanding not only of the activity of a certain drug but also what compensatory mechanisms might limit the therapeutic efficacy or promote resistance to treatment. These interactions reflect a systems-level organization of the tumor microenvironment, governed by interconnected hubs rather than linear pathways. Identifying and targeting one of these hubs, such as the IL-6IF-driven IL-6/HIF-1α/STAT3 axis, may therefore have broad therapeutic implications.

The complexity of these interrelated mechanisms was recently highlighted. Zhang et al. [161] described histone lactylation and demonstrated that increased lactylation correlated with M1 to M2 macrophage polarization, using Arg-1 as a marker of M2 macrophages. However, further analysis by Dichtl et al. [162] identified a more complex situation, where IL-6 played a major role. Furthermore, the choice of cellular model was crucial for the observed effects. This underscores the need for systematic exploration of parallel, potentially interacting mechanisms and for the use of well-standardized experimental models.

Another example illustrating the need for parallel analysis of immunoregulatory mechanisms involves the expression of MHC class II. The class II transactivator (CIITA) induces synthesis of MHC class II [163] and is regulated by the class III deacetylator SIRT1 [164]. Interestingly, SITR1 is also involved in the activity of several molecules of importance for tumor control and progression [165,166]. Consequently, multiple research projects are focusing on the development of SIRT1 inhibitors [167,168], but the risk that these inhibitors might also block CIITA and thereby down-regulate anti-tumor immune reactivity is often overlooked. Interestingly, an SIRT1 inhibitor was found to increase IL-6 production in an airway epithelial cell line [169], possibly supporting IL-6-mediated tumor-promoting activity.

In this context, it is relevant to consider the characteristics of the most efficient cancer drugs. Drugs with unique targets not influenced by parallel mechanisms, such as checkpoint inhibitors, monoclonal antibodies directed against well-defined epitopes (e.g., anti-CD19/20, anti-Her-2 and anti-EGF-antibodies), have shown remarkable therapeutic efficacy. Similarly, CAR-T cells with highly specific chimeric T-cell receptors exhibit strong therapeutic effects.

IL-6 acts as an enhancer of inflammation. Hypoxia influences numerous aspects of tumor biology, and in many of these mechanisms, IL-6 has a central inducing and regulatory role. Several signal transduction pathways of importance in tumor progression (PI3K/AKT/mTOR, Ras/MAPK, Wnt/β-catenin and Notch) are involved in mutual regulation of IL-6; that is, they are stimulated by IL-6 and stimulate IL-6 production. DAMP binding to the TLR and RAGE receptors can induce IL-6. The transcription factor NF-kappaB is a key regulator of inflammation and synthesis of IL-6, and this cytokine also has the capacity to modulate the activity of NF-kappa B. Synergistic interaction between STAT3 and NF-kappa B results in further amplification of the production of IL-6 and other cytokines, such as the IL-6 Amplifier [89]. These mechanisms create IL-6-enhancing and amplifying loops, further promoting tumor progression.

This provides a rational basis for combination treatment approaches, aiming to enhance the efficacy of immunotherapy by inhibiting IL-6. However, broad inhibition with non-specific blocking of IL-6 is complicated by the need to preserve IL-6 for the normal function of the immune system, which is required for cancer control. IL-6IF-driven pathological IL-6 production, not involving other regulatory networks, can be selectively inhibited by specific monoclonal antibodies, preserving normal IL-6–dependent immune function. Thus, based on data on the negative effects of enhanced IL-6 in immunotherapy, combining IL-6IF blockade with immune checkpoint inhibitors could synergistically restore anti-tumor immune reactivity and thereby improve immunotherapy responses.

Given the complex interaction of mechanisms promoting tumor progression and dysregulating the immune system in cancer, multiple fundamental mechanisms have been explored to find therapeutic targets. However, if one mechanism is targeted while others with similar effects remain unaddressed, outcomes are likely to be suboptimal, and resistance may emerge.

The development of new drugs has to be based on a thorough analysis to identify key regulatory hubs or combinations of these mechanisms that can be efficiently targeted. Targeting transcription factors such as STAT3 and HIF-1α with wide downstream networks has so far had limited success, likely due to functional overlap and compensatory activity among IL-6, STAT3, hypoxia and lactate. Targeting of these factors also carries the risk of interfering with essential normal cellular functions, thereby causing serious adverse events. Focusing on IL-6IF, upstream regulating of IL-6, an enhancer of multiple pro-tumor pathways, might therefore achieve broader efficacy with reduced toxicity.

In contrast to currently available IL-6 inhibitors and interacting tumor-promoting regulatory mechanisms, recombinant monoclonal antibodies directed against IL-6IF just inhibit IL-6 synthesis and thereby normalize the pathologically enhanced IL-6 concentration. No other regulatory mechanisms seem to be involved, and the physiological level of IL-6 (needed for cancer control) is left intact. IL-6 or its receptor is not blocked. This strategy will presumably result in a limited number of adverse events. Furthermore, the therapeutic anti-IL-6IF antibodies should not result in adverse events as their paratope is identical to that of autoantibodies present in healthy people.

Beyond its therapeutic importance as a target, IL-6IF can also serve as a biomarker for identifying tumors with pathologically enhanced IL-6-driven inflammation and metabolic remodeling. Quantification of IL-6IF could aid in selecting patients likely to benefit from IL-6-targeted interventions and be used to optimize therapeutic administration of anti-IL-6IF antibodies.

IL-6 has a central regulatory role in hypoxia-driven tumor-promoting mechanisms, including HIF-1α activation, angiogenesis, EMT, metastasis and lactate production. Furthermore, IL-6-induced tumor progression is promoted by several enhancing loops related to signal transduction pathways and amplifying loops related to the interaction between STAT3 and NFkB. These activities may be inhibited by selectively blocking IL-6IF, preventing pathological IL-6 production while preserving normal immune function.

## 9. Conclusions

In conclusion, these findings support the concept that cancer-associated immune dysregulation is organized around interconnected regulatory hubs, among which the IL-6IF–IL-6–HIF-1α–STAT3 axis is of central importance. Targeting IL-6IF provides, by inhibiting pathological IL-6 synthesis, a new opportunity to disrupt multiple tumor-promoting pathways while maintaining physiological immune homeostasis. Future studies should determine whether IL-6IF inhibition can restore immune surveillance, sensitize tumors to immunotherapy, and serve as a predictive biomarker of therapeutic response.

## Figures and Tables

**Table 1:** Comparison of tumor-promoting activities and mechanisms induced by IL-6 and hypoxia.

Tumor Characteristics	References	
	Hypoxia/HIF	Interleukin-6
Angiogenesis	[6,7]	[8,9]
Tumor cell proliferation	[10,11]	[12]
Metabolic reprogramming	[13,14]	[15]
Stemness	[16,17]	[18,19]
EMT	[20,21]	[22,23]
Cell invasion	[10,24]	[25,26]
ECM remodeling	[27,28]	[29,30]
Metastasis	[31,32]	[26,33]
Tumor cell survival	[34,35]	[36,37]
Immune evasion	[38,39]	[40]

## Data Availability

No new data were created or analyzed in this study.
